# Sequencing and Analysis of Lumpy Skin Disease Virus Whole Genomes Reveals a New Viral Subgroup in West and Central Africa

**DOI:** 10.3390/v16040557

**Published:** 2024-04-03

**Authors:** Ismar R. Haga, Barbara B. Shih, Gessica Tore, Noemi Polo, Paolo Ribeca, Delgerzul Gombo-Ochir, Gansukh Shura, Tsagaan Tserenchimed, Bazarragchaa Enkhbold, Dulam Purevtseren, Gerelmaa Ulziibat, Batchuluun Damdinjav, Lama Yimer, Fufa D. Bari, Daniel Gizaw, Adeyinka Jeremy Adedeji, Rebecca Bitiyong Atai, Jolly Amoche Adole, Banenat Bajehson Dogonyaro, Pradeep Lakpriya Kumarawadu, Carrie Batten, Amanda Corla, Graham L. Freimanis, Chandana Tennakoon, Andy Law, Samantha Lycett, Tim Downing, Philippa M. Beard

**Affiliations:** 1 The Pirbright Institute, Ash Road, Pirbright, Woking GU24 0NF, UK; ismar.haga@ed.ac.uk (I.R.H.); noemi.polo@pirbright.ac.uk (N.P.); paolo.ribeca@bioss.ac.uk (P.R.); carrie.batten@pirbright.ac.uk (C.B.); graham.freimanis@pirbright.ac.uk (G.L.F.); chandana.tennakoon@pirbright.ac.uk (C.T.); tim.downing@pirbright.ac.uk (T.D.); 2 The Roslin Institute, University of Edinburgh, Easter Bush Campus, Roslin, Midlothian EH25 9RG, UK; b.shih@lancaster.ac.uk (B.B.S.); andy.law@roslin.ed.ac.uk (A.L.); samantha.lycett@ed.ac.uk (S.L.); 3 Division of Biomedical and Life Sciences, Faculty of Health and Medicine, Lancaster University, Lancaster LA1 4YW, UK; 4 UK Health Security Agency, 61 Colindale Ave, London NW9 5EQ, UK; 5 NIHR Health Protection Research Unit in Genomics and Enabling Data, Mathematics Institute, Zeeman Builing, University of Warwick, Coventry CV4 7AL, UK; 6 NIHR Health Protection Research Unit in Gastrointestinal Infections, Ronald Ross Building, University of Liverpool, Liverpool L69 7BE, UK; 7 Biomathematics and Statistics Scotland, James Maxwell Clerk Building, Peter Guthrie Tait Road, Kings Buildings, Edinburgh EH9 3FD, UK; 8 Laboratory of Transboundary Animal Disease Diagnosis and Surveillance, State Central Veterinary Laboratory, Zaisan, Ulaanbaatar 17024, Mongolia; delgerzul@scvl.gov.com (D.G.-O.); gansukh@scvl.gov.mn (G.S.); tsagaan@scvl.gov.mn (T.T.); bazarragchaa@scvl.gov.mn (B.E.); dulam@scvl.gov.mn (D.P.); gerelmaa@scvl.gov.mn (G.U.); 9 General Authority for Veterinary Service, Ministry of Food, Agriculture and Light Industry, Ulaanbaatar 13381, Mongolia; batchuluun.scvl@gmail.com; 10 School of Veterinary Medicine, Wollega University, Nekemte P.O. Box 395, Ethiopia; lemayimer@gmail.com; 11 College of Veterinary Medicine and Agriculture, Addis Ababa University, Bishoftu P.O. Box 3434, Ethiopia; fufa.bari15@gmail.com; 12 Animal Health Institute (AHI), Sebata P.O. Box 04, Ethiopia; nebiyudan@gmail.com; 13 National Veterinary Research Institute, Vom 930103, Nigeria; yinkadeji@yahoo.com (A.J.A.); beckybitiyong@gmail.com (R.B.A.); adolejolly@gmail.com (J.A.A.); bbdogonyaro@gmail.com (B.B.D.); 14 Department of Animal Production and Health, Gatambe, Peradeniya 20400, Sri Lanka; pradeep_lakpriya@yahoo.co.uk; 15 School of Life Sciences, Keele University, Staffordshire ST5 5BG, UK

**Keywords:** lumpy skin disease virus, poxvirus, cattle

## Abstract

Lumpy skin disease virus (LSDV) is a member of the capripoxvirus (CPPV) genus of the *Poxviridae* family. LSDV is a rapidly emerging, high-consequence pathogen of cattle, recently spreading from Africa and the Middle East into Europe and Asia. We have sequenced the whole genome of historical LSDV isolates from the Pirbright Institute virus archive, and field isolates from recent disease outbreaks in Sri Lanka, Mongolia, Nigeria and Ethiopia. These genome sequences were compared to published genomes and classified into different subgroups. Two subgroups contained vaccine or vaccine-like samples (“Neethling-like” clade 1.1 and “Kenya-like” subgroup, clade 1.2.2). One subgroup was associated with outbreaks of LSD in the Middle East/Europe (clade 1.2.1) and a previously unreported subgroup originated from cases of LSD in west and central Africa (clade 1.2.3). Isolates were also identified that contained a mix of genes from both wildtype and vaccine samples (vaccine-like recombinants, grouped in clade 2). Whole genome sequencing and analysis of LSDV strains isolated from different regions of Africa, Europe and Asia have provided new knowledge of the drivers of LSDV emergence, and will inform future disease control strategies.

## 1. Introduction

Poxviruses are large, complex DNA viruses that replicate entirely in the cytoplasm of infected cells. Considerable effort has been put into the study of orthopoxviruses such as vaccinia virus, mpox virus and cowpox virus; however, the genus capripoxvirus (CPPV) has been relatively neglected. The CPPV genus contains three virus species: goatpox virus (GTPV), sheeppox virus (SPPV) and lumpy skin disease virus (LSDV), which cause severe disease in ruminant species. The capripoxvirus genome consists of linear, double-stranded DNA approximately 151 Kb in length, with terminal repeat regions and covalently joined “hairpin” ends forming a continuous polynucleotide chain [1]. This large and complex viral genome is predicted to encode over 150 proteins. The overall structure of the genome of capripoxviruses is similar to other poxviruses with a highly conserved core genome which encodes for proteins essential for viral replication, and a more variable accessory genome towards the periphery of the linear genome which encodes for proteins involved in virus-host interactions.

Lumpy skin disease has seen an unprecedented spread since 2012 from northern Africa and the Middle East into south-eastern Europe and, more recently, throughout Asia. This epidemic has caused substantial economic loss through loss of stock, loss of production, loss of access to international markets, and the cost of control measures [2,3]. The evolutionary history, population structure and geographic distribution of LSDV are complex with wildtype virus strains, vaccine-derived strains, and recombinant strains (a mix of both wildtype and vaccine sequence) circulating in cattle populations [4,5]. Sequencing and analysis of whole genomes of LSDV strains from a range of geographical regions and timepoints will help draw a clearer picture of the drivers for emergence of this virus, and inform decisions over the most appropriate vaccine choice and diagnostic tools. 

We used a previously published pipeline [6,7] to sequence whole genomes of LSDV from the virus archives of the Pirbright Institute, as well as recent LSDV field samples from Sri Lanka, Mongolia, Nigeria and Ethiopia. These samples cover both regions and dates previously underrepresented and provide novel information on the phylogeography of LSD, specifically in documenting new lineages in west and central Africa.

## 2. Materials and Methods

### 2.1. Cells and Viruses

Viruses were grown in Madin-Darby Bovine Kidney (MDBK) cells (ATCC CCL-22), as described previously [6]. Viruses were obtained from the Pirbright Institute virus archives or via submissions to the World Organisation for Animal Health (WOAH) lumpy skin disease virus reference laboratory at the Pirbright Institute (Table 1).

### 2.2. Purification of LSDV DNA 

LSDV was purified using a protocol adapted from [8] and described previously [6]. MDBK cells were infected with LSDV and incubated for 7 d. Cells were then scraped into supernatant, and samples centrifuged at 2000 RPM for 10 min. Cell pellets were resuspended in 20 mL of 1 mM Tris-HCl pH9 and sonicated 3 times for 1 min in a cup horn sonicator at approximately 160 W. Five µL of Benzonase^®^ (>250 units/µL, Sigma E1014-25KU, Gillingham, UK) were added to the samples, followed by incubation for 30 min at RT and centrifugation at 2000 RPM for 10 min. Supernatants were collected and layered on 5 mL of a 36% sucrose solution in 1 mM Tris-HCl pH9 in an OptiSeal tube (Beckman, High Wycombe, UK), followed by centrifugation at 13,500 RPM for 80 min in a SW-28 rotor (Beckman). Supernatants were discarded, pellets resuspended in 3 mL of 1 mM Tris-HCl pH9 and sonicated as above before being layered on 1 mL of 36% sucrose in 1 mM Tris-HCl pH9 in an OptiSeal tube (Beckman). Samples were centrifuged at 15,700 RPM for 80 min in a SW-55 rotor (Beckman). The pellets were resuspended in 500 µL of 1 mM Tris-HCl pH9 in a nuclease-free tube. Samples were then treated with 33 µL of 1.5 M Tris pH 8.8, 50 µL of 10% SDS, 100 µL of 60% sucrose and 85 µL of proteinase K (20 mg/mL, Thermo Fisher Scientific, Altrincham, UK) for 4 h at 37 °C, followed by phenol-chloroform extraction and ethanol precipitation.

### 2.3. Whole Genome Sequencing (WGS)

Samples were quantified using the Qubit dsDNA High Sensitivity kit and diluted to 0.2 ng/µL for library preparation for a total DNA input of 1 ng. Libraries were generated using the Nextera XT DNA library preparation kit (Illumina, San Diego, CA, USA) on the Hamilton NGSStar (Hamilton Robotics, Bonaduz, Switzerland) following the manufacturer’s instructions. Libraries were quality-checked using the Agilent 4200 Tapestation with D1000 High Sensitivity reagents prior to bead normalisation. Following bead normalisation, all libraries were paired and sequenced (2 × 150 bp) on the Illumina Miseq using v2 reagents and a 1% PhiX spike in (Illumina). This produced a median of 303,308 ± 241,842 (±standard deviation) reads per sample. After filtering, this was a median of 114,134 (±105,886) reads per sample, which generated a median average read-depth 203-fold (±216) (Table S1).

### 2.4. Genome Assembly and Mutation Detection

A hybrid genome assembly approach was applied that integrated de novo assembly for the core genome and read mapping for the 5′ and 3′ end accessory genomes [7]. This was to mitigate the effects of high recombination rates at the accessory genome regions relative to the conserved core genome region. The core genome was a conserved 93,060 bp region between bases 13,851 (the start of gene LD020, encoding a ribonucleotide reductase small subunit) and 106,910 (before gene LD116, encoding an RNA polymerase subunit) bases (inclusive). The 5′ accessory genome began at base 2500 (the first coding region) to base 13,850 (13,610 bp in total). The 3′ accessory genome was designated from bases 106,911 to 148,000 (the end of the last coding region) (43,645 bp in total).

The core genome de novo assembly used SPAdes v3.13.2 [9] to generate initial assemblies using k-mer sizes of 33, 55, 77, 85 and 99. These contigs were scaffolded on the reference LSDV genome 155920/2012 isolated in Israel (KX894508, 150,562 bp) with RaGOO v1.1 [10] to produce an assembly with an average length of 150,565 bp (range 150,502 – 150,642 bp) for each of the 21 samples. To resolve the accessory genome regions, each library’s reads were mapped to this reference with BWA-MEM v0.7.17 [11]. The SAM files were processed with SAMtools v1.10 [12] and mutation screening was implemented with FreeBayes v-1.3.1 [13]. The read-mapping consensus genome per sample was created using BCFtools v1.10 for single nucleotide polymorphisms (SNPs) with %QUAL ≥ 20 and sufficient read depth, as ascertained by mosdepth v0.2.5- [14]. The latter consensus sequences were compared to the de novo assemblies using Minimap2 v2.16 [15] ensuring that mutations from the de novo assembly were favoured for retention in the resulting hybrid assembly. These comprised 4655 transitions (Ts) and 1454 transversions (Tv) (Ts/Tv = 3.2), which was lower at the 5′ (Ts/Tv = 184/75 = 2.5) and 3′ (Ts/Tv = 597/216 = 2.8) ends. The core genome had five triallelic sites, the 5′ end had one such site and the 3′ end had four triallelic sites.

### 2.5. Genome Annotation, Diversity and Clustering

The 21 hybrid assemblies were annotated using Prokka [16] for the *Capripoxvirus* genus using the reference genome annotation that had 163 CDS regions. This reference was retained in subsequent analyses for context, resulting in 22 sequences. The virtual environments and packages used for these analyses were managed by Conda v22.9.0 (Anaconda Software Distribution 2022). A subset of analyses was implemented on n = 18 samples that excluded the Hong Kong, Neethling and both Mongolia samples; these four were genetically distinct from the others. The 22 sequences were aligned using MUSCLE v3.8.1551 [17]. The SNPs were extracted per sample as Variant Call Formats (VCFs) using SNP-sites [18]. Comparisons of sequence diversity across groups and between sample pairs was implemented with VCF-kit [19]. To find genetic groups among the 22 samples, we assessed the SNP diversity in the whole, core and 5′ and 3′ accessory genomes as sparse matrices using a hierarchical Bayesian clustering algorithm implemented in Fastbaps v1.0.8 (Fast Hierarchical Bayesian Analysis of Population Structure, [20]) in R v4.1.1 [21] and RStudio v2022.02.3 [22] with packages ape v5.6-2 [23], devtools v2.4.4 (Wickham et al. 2022), ggplot2 v3.3.6 [24], ggtree v3.2.1 [25] and phytools v1.2-0 [26]. This used default parameters and a Dirichlet prior variance independently estimated for each of the four datasets.

### 2.6. Phylogenetic, Temporal and Recombination Analysis

Phylogenetic reconstruction of sample evolutionary relationships for the groups of 18 and 22 samples was conducted using RAxML (Randomised Axelerated Maximum Likelihood) v8.2.12 with a GTR (general time reversible) substitution model [27]. This was implemented for the whole genome, core genome, 5′ accessory region and 3′ accessory region. These phylogenies were mid-pointed rooted and visualised using FigTree v1.4.4 [28] and phangorn v2.10.0 [29], treeio v1.18.1 [30] and phytools v1.2-0 [26] in RStudio. Although the reference was sequenced using different sequencing approaches (PacBio and capilliary) compared to that used for the 21 samples (Illumina), no substantive artefacts in the phylogenies were evident. The correlation of genetic distance to the phylogeny roots with time since isolation was examined for the 11 samples with known isolation dates using TempEst v1.7 [31]. Recombination patterns across the 150 Kb genome were examined across 9240 pairwise comparisons possible in the set of 22 samples using 3Seq v1.7 [32] with an 8 g *p*-value table; this was repeated separately for the core, 5′ accessory and 3′ accessory genomes. 3Seq uses a conservative Dunn–Sidak *p* value correction to adjust for multiple testing [32], and was also used to measure genetic diversity per region using the nucleotide diversity (Pi) and Watterson and Wu’s theta [33]. At the 5′ accessory region, three sample pairs were genetically identical (Ethiopia2-Ethiopia1, SriLanka1-SriLanka3 and Mongolia1-Mongolia2). At the 3′ end, the four Ethiopia samples were genetically identical, as were SriLanka2 and SriLanka3. Consequently, 18 samples were examined at the 5′ and 3′ regions. Code and data to create these analyses are available at https://github.com/downingtim/LSDV_Africa/. Last accessed: 21 December 2023.

## 3. Results

Full genome sequencing and analysis of a unique set of 21 LSDV strains from diverse geographic sources, including Africa and the Middle East, and diverse ages was carried out (Table 1). The analysis included strains from recent outbreaks in Sri Lanka, Ethiopia, Nigeria and Mongolia. 

LSD in Sri Lanka. On 9 September 2020, the first case of LSD in Sri Lanka was reported in Northern Province in Kopay Veterinary Range in Sirupiddy East village in Jaffna District. LSD is a vector-borne disease and climatic conditions of Sri Lanka are favourable for the propagation of vectors; therefore, the disease spread progressively further throughout the country. A total of 24,146 cases and 102 deaths were reported in 2020 and 8241 cases reported in 2021.

LSD in Ethiopia. Between 2018 and 2019 LSD, outbreaks were identified in three districts of Ethiopia: Tiyo and Munessa in October and November 2018, Bishoftu in January 2019, and Oda Bultum in March and April 2019. All suspected cases exhibited characteristic skin nodules. Out of 242 examined animals, 14 (5.8%) cattle were clinically infected, showing signs such as fever, nasal discharges, depression, and skin nodules. The animal level morbidity in affected cattle was 5.8%, with a case fatality rate of 21.4%.

LSD in Nigeria. The sequenced sample was collected on the 21 September 2019, in Bokkos, Plateau State, Nigeria from a 5-year-old White Fulani breed of Nigerian indigenous cattle. The animal was in a herd of 110 cattle. The clinical signs observed were fever, generalized nodular skin lesions, edema of the legs and brisket, and lymphadenopathy. Herd morbidity was 18.18% (20/110) and mortality 4.54% (5/110).

LSD in Mongolia. LSD was first detected in Mongolia in 2021 in the eastern provinces of Dornod and Sukhbaatar. The outbreak is described in [34]. Samples from the skin of diseased cattle were collected and sent to the Pirbright Institute for full genome sequencing and analysis.

In addition to LSDV strains from recent and historical field outbreaks, we also sequenced two “vaccine” strains of LSDV from the Pirbright archives—a LSDV Neethling strain and a LSDV KS-1 strain. The LSDV strain Neethling was generated in 1959 by the serial passage of a field strain in lamb kidney cells and chorioallantoic membranes of embryonated hens’ eggs [35]. The KS-1 strain is a vaccine strain derived from the KSGP O-240 vaccine strain generated by a serial passage in Kenya in the 1970s [36]. The sequence of Pirbright Neethling differed at 314 sites compared to the Neethling vaccine LW 1959 isolate (AF409138), corresponding to 99.80% similarity, and the sequence of Pirbright KS-1 differed at 74 sites compared to the KSGP O-240 sample (KX683219.1), corresponding to 99.95% similarity.

To investigate the evolutionary origins of the field samples, we examined their genomic variability to evaluate their diversity, population structure, ancestry and recombination patterns. We have used the terminology for naming strains proposed by Biswas and colleagues [1] and subsequently used by others [4,35]. The Neethling-like viruses form clade 1.1, and the Kenya-like viruses and wildtype viruses clade 1.2. Vaccine-like recombinant strains are classed in clade 2. Including a reference genome from clade 1.2 for context, we examined 22 samples: one from clade 1.1, 18 from clade 1.2, and three from clade 2.

### 3.1. Elevated SNP Diversity at the 5′ and 3′ Accessory Genome Regions

We focused on the genetic patterns within the conserved 93.1 Kb core genome (at bases 13,851–106,910), the 11.35 Kb 5′ accessory genome (bases 2500–13,850), and the 41.1 Kb 3′ accessory genome (bases 106,911–148,000). The core genome had 939 SNPs (10.1 SNPs/Kb), whereas the 5′ (260 SNPs, 22.9 SNPs/Kb) and 3′ (816 SNPs, 19.9 SNPs/Kb) ends had higher SNP densities (Table 2). There were 2015 genome-wide SNPs (13.8 SNPs/Kb), and the mean number of SNPs between sample pairs was 462. The higher rate of theta/Kb compared to Pi/Kb across the genome indicated a higher rate of low-frequency SNPs (Table 2). This was driven by population structure because these metrics were more equal (as expected) when computed for clade 1.2, only without the genetically distinct clade 1.1. 

### 3.2. Model-Based Classification Finds Four Genetic Groups

**Figure 1:**
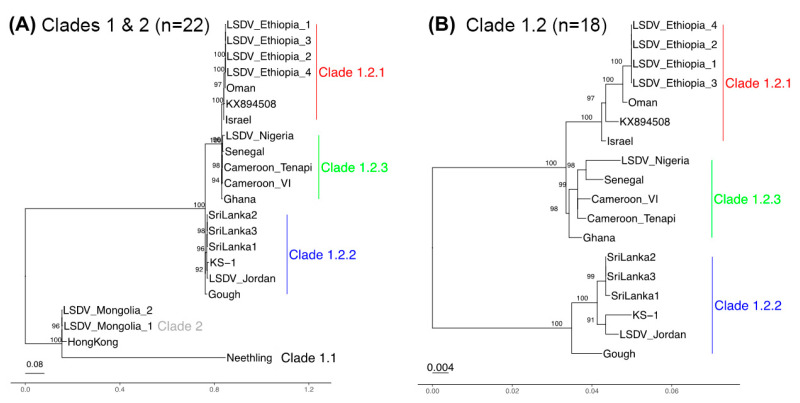
Core genome phylogenies for (**A**) all 22 LSDV samples and (**B**) clade 1.2 only (18 samples). (**A**) The clade 1.1 Neethling strain was distinct from the clade 2 Hong Kong/Mongolia isolates. (**B**) Clade 1.2 was divided into three groups: clade 1.2.2 related to Kenyan sheep and goat pox (KSGP) vaccines (blue, n = 6), clade 1.2.3 linked to west and central Africa (green, n = 5) and clade 1.2.1 with samples from the Middle East and east Africa (red, n = 7). Bootstraps with values > 90 are shown.

The 22 strains separated into clades 1.1, 1.2, and clade 2 based on clustering with Fastbaps, as expected (Figure S1A). The Neethling strain clustered in clade 1.1. Three strains clustered in clade 2 (Hong Kong, Mongolia M2_S5 and Mongolia M5_S6) (Figure 1A). The remaining 18 samples clustered within clade 1.2 (Figure 1A,B). These 18 samples could be further differentiated into three subgroups. Six samples from Israel, Oman and Ethiopia grouped with the reference strain from clade 1.2 (subgroup 1.2.1). Six samples grouped together in subgroup 1. 2.2, often referred to as “Kenya-like” (LSDV_Jordan, LSDV-Gough, LSDV_Sri Lanka 1–3 and KS-1), and five samples originating from four countries in central and west Africa (Ghana, Cameroon, Nigeria and Senegal) grouped together in the newly named subgroup 1.2.3. These sequences were particularly interesting as they have not been reported previously, and, therefore, reflect a layer of diversity unsampled until this study.

### 3.3. Phylogenetic Separation between and within Clades 1.1 and 1.2

Our phylogenetic reconstruction of the evolutionary relationships confirmed the five genetic groups (clade 1.1, 1.2.1, 1.2.2, 1.2.3 and clade 2). We focused initially on the 93 Kb core genome as the most informative representation of their collective ancestries, at which clades 1.1 and 1.2 differed by 951 SNPs in total. The core genome phylogeny showed that the clade 2 Hong Kong/Mongolia trio were different from clade 1.1 Neethling because the latter had 801 unique SNPs (Figure 1A), though Neethling was somewhat less distinct at the 5′ region (Figure S2). Within clade 1.2, 31 SNPs differentiated clade 1.2.1 (n = 6 samples: Sri Lanka 1/2/3, KS-1 isolated in Kenya, LSDV_Jordan and Gough) from clades 1.2.2 and 1.2.3 (n = 12 samples, isolated from Africa and the Middle East) (Figure 1B). This was consistent across the genome: two of the 33 SNPs were at the 5′ region (0.22 SNPs/Kb), 15 were at the core (0.16 SNPs/Kb) and 14 were at the 3′ region (0.34 SNPs/Kb) (Figure S3). Within the clade 1.2.2, there was one core genome SNP and one 3′ end SNP distinguishing the Sri Lankan samples from LSDV Jordan, LSDV KS-1, and LSDV Gough. Within this group, LSDV Gough had higher differentiation at its 5′ region relative to the core and 3′ end (Figure S2C). The correlation between root-to-tip distance and genome-wide variation over time for the 11 taxa with known isolation dates was small and had no meaningful signal (r = 0.18, r^2^ = 0.032, via Tempest v1.7) (Rambaut et al. 2016). This indicated the absence of a molecular clock signal in these samples, indicating discontinuity in the mutation rate or generation times. Our lack of additional dated samples could not inform this further.

### 3.4. A Genetically Distinct West and Central Africa Subgroup

**Figure 2:**
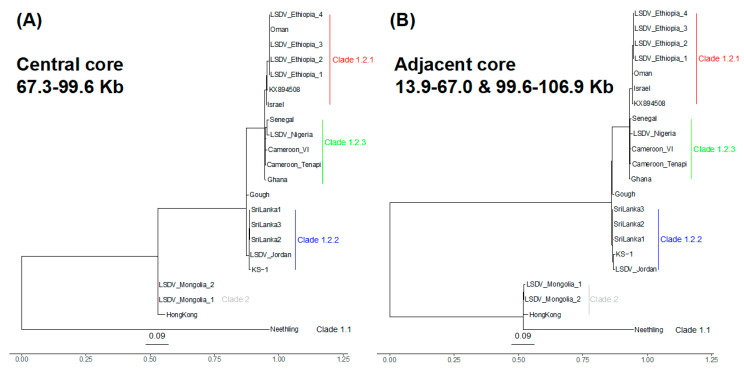
Phylogenies of the (**A**) central (67,250 to 99,575 bp) and (**B**) adjacent core genome regions (13,851–66,966 and 99,608–106,910 bp, concatenated). (**A**) At the central core genome, clade 2’s Hong Kong and the Mongolia pair (grey) had ancestry more like clade 1.2.2 (clade 1.2.2 in blue, clade 1.2.3 in green, clade 1.2.1 in red). (**B**) At the adjacent core genome regions, clade 2 was more like Neethling (clade 1.1).

The clade 1.2.1 (n = 7 samples: reference genome KX849508, the four Ethiopian samples, Oman and Israel) differed from clade 1.2.3 (n = 5 samples: Cameroon_Tenapi, Cameroon_VI, Ghana, Nigeria, Senegal) by 29 SNPs (Figure 2B). This was a genome-wide trend: four of the 29 SNPs were at the 5′ region (0.35 SNPs/Kb), 15 were at the core (0.16 SNPs/Kb) and ten at the 3′ region (0.23 SNPs/Kb) (Figure S3). This demonstrated that clade 1.2.3 had distinctive ancestries and, thus, was a novel finding. Notably, clade 1.2.3 had no shared ancestral SNPs at the 5′ region, unlike the core and 3′ accessory genomes (Figure S3C), implying high variability within this undersampled set of lineages. Within clade 1.2.3, there was one core genome SNP and one 3′ accessory genome SNP between the four Ethiopian samples and the samples isolated in Oman and Israel (Figure S3).

### 3.5. Recombinant Origins of Hong Kong and the Mongolia Samples

The genome-wide variation in ancestry patterns pointed to recombination as a force shaping the genetic composition of these samples. All of the clade 2 samples and 8 of the clade 1.2 samples showed evidence of recombination. At the core region, the sole recombination breakpoint was in the sequence of the Hong Kong isolate and the Mongolian pair of isolates. This recombinant region reflected ancestry more similar to clade 1.2.2 than Neethling and had a length of 32 Kb (at 66,966–67,250 to 99,575–99,608 bp). It spanned 41 CDSs, from the 3′ end of LD075 (encoding a RNA polymerase-associated protein) to the 5′ end of LD105 (encoding an IMV membrane protein). The evolutionary origins of the genetic variation for the Hong Kong and Mongolia pair were quite distinct for this central 32 Kb core region compared to the adjacent 55 Kb core genome regions, consistent with a different origin from an unsampled lineage distantly related to clade 1.2.2 (Figure 2). Additionally, there was further evidence that the Hong Kong and Mongolia samples were recombinants; those three samples had an 8.8 Kb 3′ accessory genome recombinant region at bases 135,013–135,261 to 144,179–144,386 (spanning LD143, encoding a tyrosine protein kinase, to LD149) that had the same pattern as this 32 Kb central core genome segment (Figure S4). Beyond this, we observed nine recombinations at five distinct tracts at the 5′ region involving samples from clades 1.1, 1.2.2 and 1.2.3. These encompassed bases 3180 to 4859 bp (at LD006 encoding an IL-1-like protein, or LD007, or LD008 encoding a soluble interferon gamma receptor) and terminated at bases 13,773 to 13,785 bp (after LD019b, encoding a kelch-like protein).

## 4. Discussion

LSD has spread rapidly throughout the Middle East, south-east Europe and Asia in the past 10 years, causing substantial loss to affected cattle industries. Our understanding of the strains of LSDV responsible for this LSD epidemic has improved in recent years with the publication and analysis of full genomes of LSDV strains isolated from the field and used as vaccines. LSDV strains isolated from the Middle East and south-east Europe since 2010 have grouped together in clade 1.2.1, while LSDV Neethling-attenuated vaccine strain along with LSDV field isolates from LSD outbreaks in South Africa in the 1950s and 1970s belong to clade 1.1 [35]. LSDV isolates with evidence of recombination between field strain and vaccine strains were first identified in Russia and then in countries in Asia, including China, Vietnam, Hong Kong and Taiwan [7,37,38,39,40]. These recombinant strains are most likely a result of a poor vaccine production technique and quality control [41], and have been grouped into clade 2 [4]. Note that some publications refer to clade 2 as group R or R4 [5].

**Figure 3:**
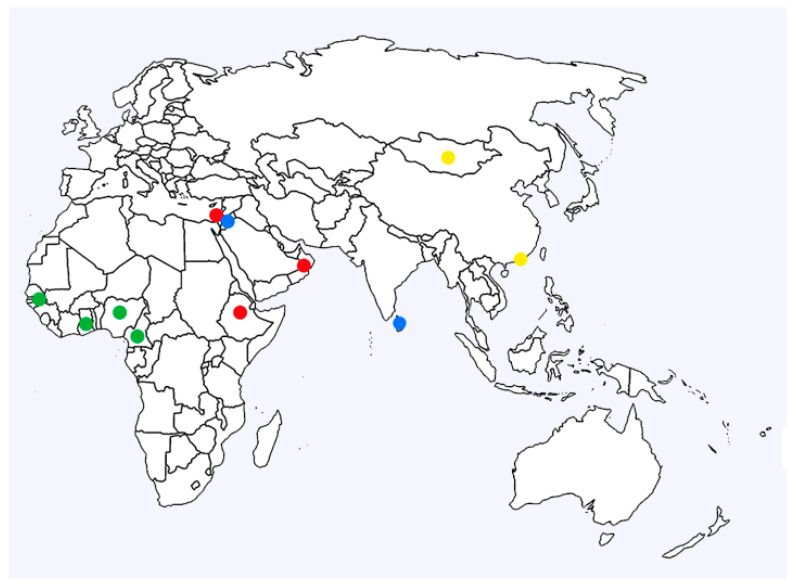
Geographic distribution of the LSDV strains. Colour designations: clade 1.2.1 red, clade 1.2.2 blue, clade 1.2.3 green, and clade 2 yellow.

Sequencing and analysis of five LSDV strains from west and central Africa revealed a previously unknown subgroup in clade 1.2, which we named clade 1.2.3 (Figure 3). The geographic clustering of the clade 1.2.3 samples and their heterogenous population structure suggests that this is an undersampled branch of the LSDV tree, with additional undiscovered lineages possibly circulating in the region. The comparative virulence of virus strains from this subgroup is unknown. The strains were isolated from outbreaks in 1997 (Senegal), 2006 (Ghana and Cameroon) and 2021 (Nigeria), suggesting the long-term circulation of this strain in the region. Interestingly, an isolate of LSDV from Nigeria from 2018 has been described previously [42]. It clustered in clade 1.2.1 along with strains from the Middle East and southeast Europe and demonstrated similar virulence in an experimental model of LSD. This indicates that multiple strains of LSDV are circulating in Nigeria.

Five isolates sequenced in this study (LSDV Gough, LSDV Jordan and the three strains of LSDV from Sri Lanka) clustered with the LSDV KS-1 strain. The KS-1 strain is part of a group of LSDV vaccines developed in Kenya, and also includes KSGP O-240 [1]. These vaccines were originally developed in Kenya to control LSD; however, multiple reports suggest they are insufficiently attenuated, do not provide strong protection, and should not be recommended for use in cattle [36,43,44,45]. The isolation of this strain from Sri Lanka is consistent with the sequence analysis of LSDV from disease outbreaks in neighbouring countries, including India, Bangladesh and Myanmar [46,47,48]. This indicates that LSD outbreaks in these countries may have a common ancestor that is distinct from the one causing outbreaks in China, Hong Kong, Mongolia and Vietnam, and suggests that LSDV from multiple origins are responsible for the current spread of LSD in Asia. It also raises the possibility that the source of LSDV in these countries could be poor vaccine choice. The Jordanian isolate clusters within clade 1.2.3, unlike most other strains originating from the Middle East, including the isolates from Israel and Oman, reported in this study. The source of this particular outbreak in Jordan remains unclear, though the use of vaccines of unknown provenance has been reported in the country [49]. 

The sequencing and analysis of whole genomes of historic and recent LSDV strains in this and other published work have revealed important information about the routes of introduction of LSDV to new regions. It has also highlighted the importance of quality control when generating and selecting vaccines for use in the field. We agree with the recommendation of others [4,5] who advocate for the use of whole genome sequencing rather than a small number of genes or markers for phylogeny reconstruction. WGS enables the precise identification of strains and reduces the likelihood of misassigning a sequence.

## Figures and Tables

**Table 1:** LSDV strains sequenced in this study.

Isolate Name	Abbreviation	Origin	Year ^1^	Acc Number
LSDV/Gough/1959	LSDV Gough	Unknown	1959	SRR27563725
LSDV/Senegal/1997	LSDV Senegal	Senegal	1997	SRR27563735
LSDV/CameroonVI/2006	LSDV Cameroon VI	Cameroon	2006	SRR27563734
LSDV/Ghana/2006	LSDV Ghana	Ghana	2006	SRR27563732
LSDV/Israel/2007	LSDV Israel	Israel	2007	SRR27563731
LSDV/Ethiopia7_3/2019	LSDV Ethiopia 4	Ethiopia	2019	SRR28085235
LSDV/Ethiopia3_2/2019	LSDV Ethiopia 3	Ethiopia	2019	SRR28085236
LSDV/Ethiopia1_1/2019	LSDV Ethiopia 1	Ethiopia	2019	SRR28085238
LSDV/Ethiopia21_4/2019	LSDV Ethiopia 2	Ethiopia	2019	SRR28085237
LSDV/HongKong/2020 ^2^	LSDV Hong Kong	Hong Kong	2020	See ref [7]
LSDV/SriLanka1/2021	LSDV Sri Lanka 1	Sri Lanka	2020	SRR27563730
LSDV/SriLanka2/2021	LSDV Sri Lanka 2	Sri Lanka	2020	SRR27563729
LSDV/SriLanka3/2021	LSDV Sri Lanka 3	Sri Lanka	2020	SRR27563728
LSDV/MongoliaM5_S6/2021	LSDV Mongolia 1	Mongolia	2021	SRR28085241
LSDV/MongoliaM2_S5/2021	LSDV Mongolia 2	Mongolia	2021	SRR28085240
LSDV/Nigeria_Bokkos_S10/2021	LSDV Nigeria	Nigeria	2021 ^3^	SRR28085239
LSDV/KS_1/1995	LSDV KS-1	Kenya	1995	SRR27563727
LSDV/Oman/2009	LSDV Oman	Oman	2009	SRR27563726
LSDV/Jordan/2013	LSDV Jordan	Jordan	2013	SRR27563724
LSDV/CameroonTenapi/UK	LSDV Cameroon Tenapi	Cameroon	Unknown	SRR27563723
LSDV/Neethling/UK	LSDV Neethling	Unknown	Unknown	SRR27563733

^1^ Year of sample arrival at TPI. ^2^ Previously reported in [7]. ^3^ Sample was received at TPI in 2021 but the collection date was recorded as September 2019.

**Table 2:** Summary statistics of genome diversity. Pi is the nucleotide diversity (the mean number of pairwise SNPs). Theta was calculated as Watterson and Wu’s theta.

Region	Length	#SNPs	SNPs/Kb	Mean Number of Pairwise SNPs	Theta/Kb	Pi/Kb
Whole genome	150,562	2023	13.5	452	4.5	3.0
5′ accessory region	11,350	260	22.9	60	7.4	5.3
Core genome	93,060	939	10.1	213	3.3	2.3
3′ accessory region	41,090	816	19.9	174	6.4	4.2

## Data Availability

Genome assemblies at https://doi.org/10.6084/m9.figshare.24121080, accessed on 21 December 2023. Code and data to create the analyses are available at https://github.com/downingtim/LSDV_Africa/.

## Supplementary Materials

The following supporting information can be downloaded at: https://www.mdpi.com/article/10.3390/v16040557/s1, Table S1. Sequencing data. Figure S1. Model-based genetic clustering. Figure S2. Phylogenies constructed for all n = 22 LSDV samples. Figure S3. Phylogenies for the n = 18 Clade 1.2 samples. Figure S4. Phylogeny of an 8.8 Kb recombination tract (at 135,261 to 144,179 bp).
