# Supplementary material for: Sequencing and Analysis of Lumpy Skin Disease Virus Whole Genomes Reveals a New Viral Subgroup in West and Central Africa
Source: Viruses. 2024 Apr 3;16(4):557. doi: 10.3390/v16040557 (PMC11053774; doi:10.3390/v16040557)
Supplement: Supplementary file 1 [file viruses-16-00557-s001.zip › viruses-2779993-supplementary.pdf]

Table S1. Sequencing data.

Sample	Read pair count (raw)	Read pair count (QC filtered and mapped)	Covered percent	Average coverage
LSDV Cameroon VI	303308	152406	99.995	303.0321
LSDV KS-1	498437	236045	99.995	474.1635
LSDV Ghana	455049	97533	99.995	186.576
LSDV Gough	519435	385538	99.995	794.2664
LSDV Israel	462304	261050	99.995	519.3423
LSDV Jordan	334547	230174	99.995	464.1506
LSDV Oman	293367	187436	99.995	371.709
LSDV Cameroon Tenapi	743667	22841	99.918	24.8808
LSDV Hong Kong	19011	10148	99.991	25.845
LSDV Sri Lanka 1	289267	196270	99.995	319.8821
LSDV Sri Lanka 2	340211	240636	99.995	397.1682
LSDV Sri Lanka 3	175445	114134	99.995	202.5817
LSDV Neethling	171577	89928	99.958	202.3082
LSDV Senegal	138969	94250	99.945	142.0289
LSDV Nigeria	536394	322308	99.995	313.543
LSDV Ethiopia 1	478946	433312	99.995	389.073
LSDV Ethiopia 2	582162	474974	99.995	465.16
LSDV Ethiopia 3	499906	427060	99.995	418.135
LSDV Ethiopia 4	369422	303582	99.979	297.148
LSDV Mongolia 1	1230424	681090	99.989	647.04
LSDV Mongolia 2	903474	577720	99.991	551.95

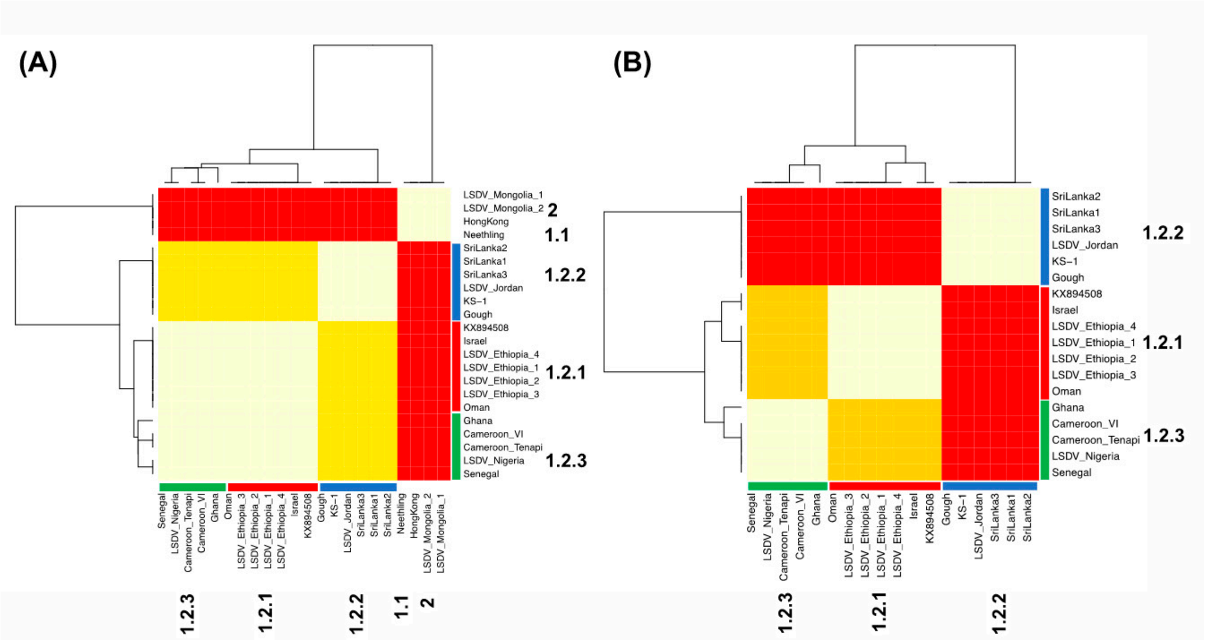

Figure S1. Model-based genetic clustering of (A) all 22 samples and (B) Clade 1.2 (18 samples) shown by the dashed box in (A). (A) Clades 1.1 and 2 were distinct, as was Clade 1.2.2 to a lesser extent. (B) Clade 1.2 was divided into three differentiated groups: Clade 1.2.2 related to Kenyan sheep and goat pox (KSGP) vaccines (blue, n = 6), Clade 1.2.3 linked to west and central Africa (green, n = 5) and Clade 1.2.1 with samples from the Middle East and Africa (red, n = 7).

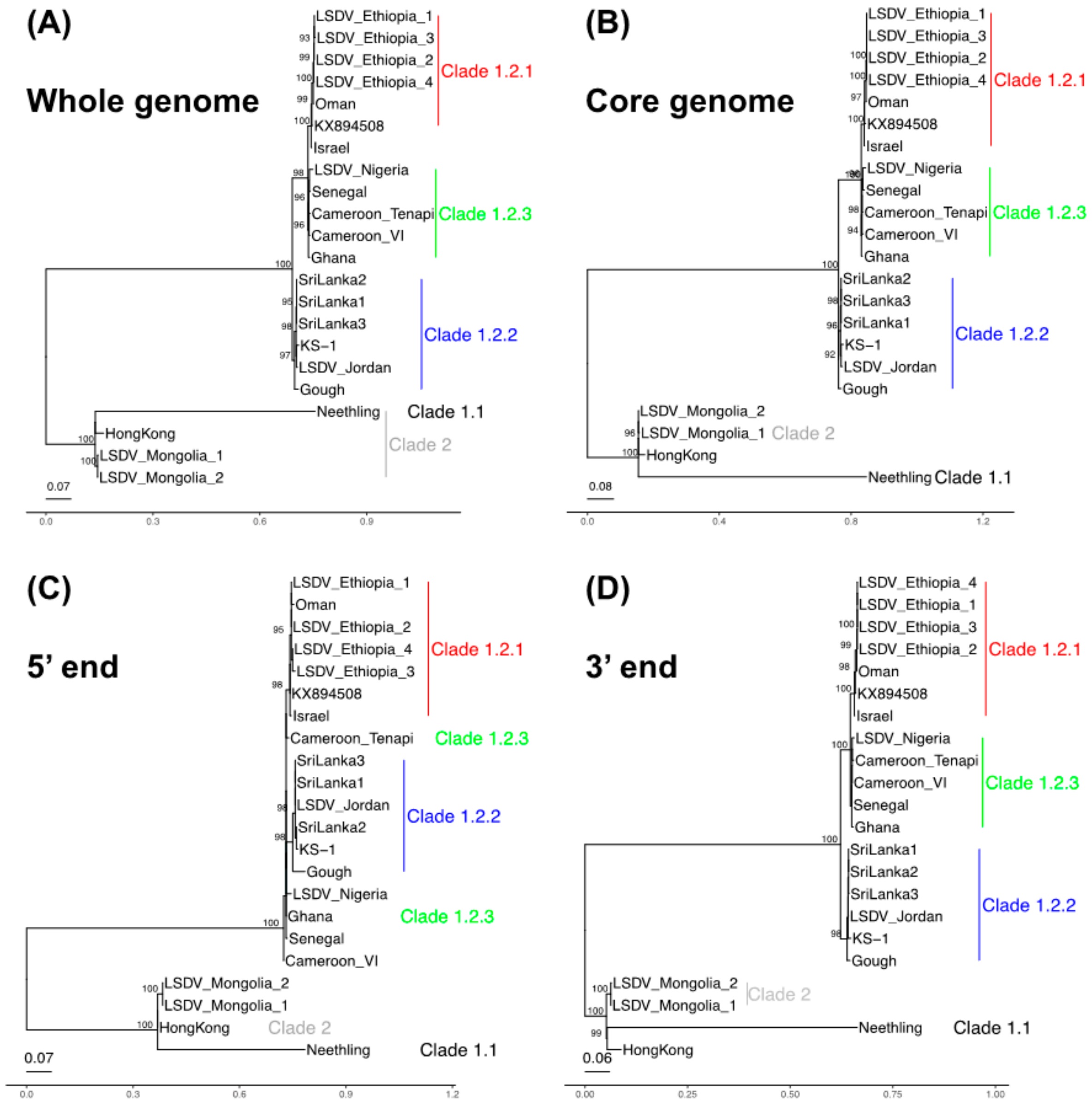

Figure S2. Phylogenies constructed for all n = 22 LSDV samples from SNPs across the: (A) whole genome, (B) core genome, (C) 5′ accessory region and (D) 3′ accessory region. Clade 1.1 (the Neethling strain) was distinct from the Hong Kong/ Mongolia group. Clade 1.2 was divided into three groups: Clade 1.2.2 related to Kenyan sheep and goat pox (KSGP) vaccines (blue, n = 6), Clade 1.2.3 linked to west and central Africa (green, n = 5) and Clade 1.2.1 with samples from the Middle East and Africa (red, n = 7). (C) The 5′ region was more divergent between Clades 1.1 and 1.2, (D) whereas the 3′ region had the opposite pattern. Bootstraps with values > 90 are shown.

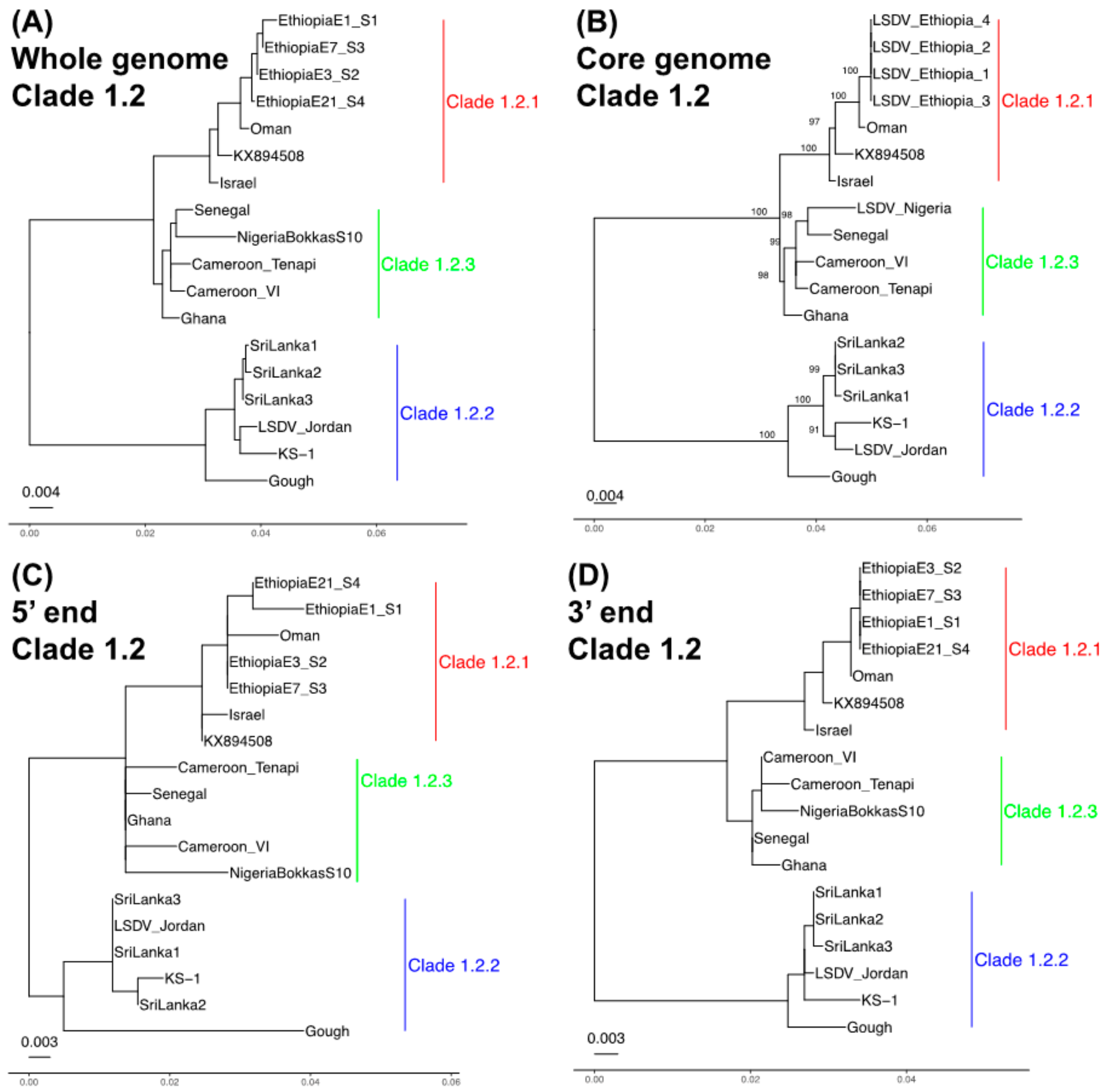

Figure S3. Phylogenies for the n = 18 Clade 1.2 samples from SNPs across the: (A) whole genome, (B) core genome, (C) 5′ accessory region and (D) 3′ accessory region. Genetic differences were lowest at the 5′ end. Clade 1.2 was divided into three groups: Clade 1.2.2 related to Kenyan sheep and goat pox (KSGP) vaccines (blue, n = 6), Clade 1.2.3 linked to west and central Africa (green, n = 5) and Clade 1.2.1 with samples from the Middle East and Africa (red, n = 7). Bootstraps with values > 90 are shown.

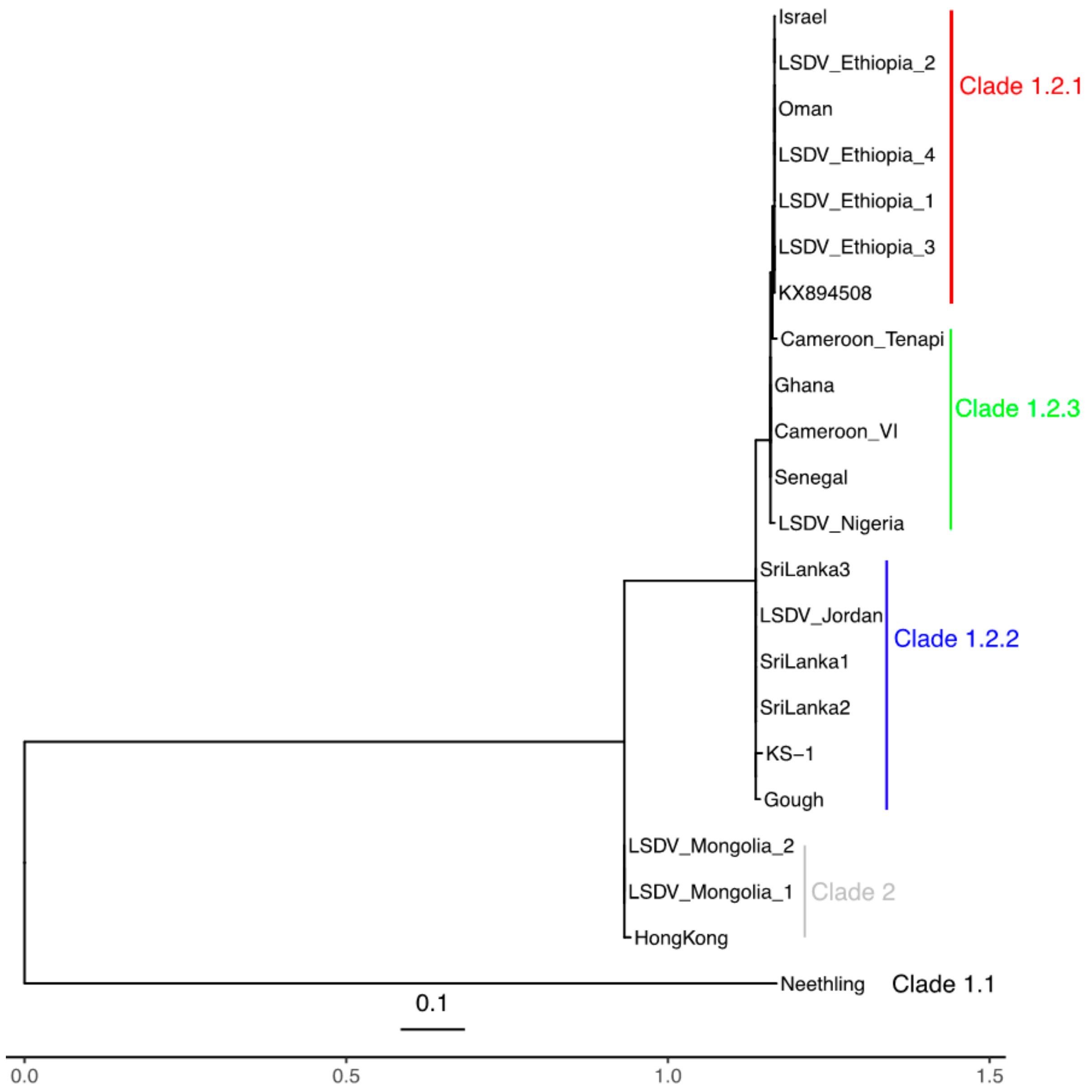

Figure S4. Phylogeny of an 8.8 Kb recombination tract (at 135,261 to 144,179 bp) shows that Clade 2's Hong Kong and Mongolia pair had ancestries more akin to Clade 1.2.2 than to Neethling in Clade 1.1. Bootstraps with values > 90 are shown.
